# Multilocus sequence analysis of *Thermoanaerobacter* isolates reveals recombining, but differentiated, populations from geothermal springs of the Uzon Caldera, Kamchatka, Russia

**DOI:** 10.3389/fmicb.2013.00169

**Published:** 2013-06-21

**Authors:** Isaac D. Wagner, Litty B. Varghese, Christopher L. Hemme, Juergen Wiegel

**Affiliations:** ^1^Department of Microbiology, University of GeorgiaAthens, GA, USA; ^2^Department of Botany and Microbiology, University of OklahomaNorman, OK, USA; ^3^Institute for Environmental Genomics, University of OklahomaNorman, OK, USA

**Keywords:** multilocus sequence analysis, *Thermoanaerobacter*, microbial biogeography, Uzon Caldera, Kamchatka, hot springs

## Abstract

Thermal environments have island-like characteristics and provide a unique opportunity to study population structure and diversity patterns of microbial taxa inhabiting these sites. Strains having ≥98% 16S rRNA gene sequence similarity to the obligately anaerobic *Firmicutes Thermoanaerobacter uzonensis* were isolated from seven geothermal springs, separated by up to 1600 m, within the Uzon Caldera (Kamchatka, Russian Far East). The intraspecies variation and spatial patterns of diversity for this taxon were assessed by multilocus sequence analysis (MLSA) of 106 strains. Analysis of eight protein-coding loci (*gyrB, lepA, leuS, pyrG, recA, recG, rplB*, and *rpoB*) revealed that all loci were polymorphic and that nucleotide substitutions were mostly synonymous. There were 148 variable nucleotide sites across 8003 bp concatenates of the protein-coding loci. While pairwise *F*_ST_ values indicated a small but significant level of genetic differentiation between most subpopulations, there was a negligible relationship between genetic divergence and spatial separation. Strains with the same allelic profile were only isolated from the same hot spring, occasionally from consecutive years, and single locus variant (SLV) sequence types were usually derived from the same spring. While recombination occurred, there was an “epidemic” population structure in which a particular *T. uzonensis* sequence type rose in frequency relative to the rest of the population. These results demonstrate spatial diversity patterns for an anaerobic bacterial species in a relative small geographic location and reinforce the view that terrestrial geothermal springs are excellent places to look for biogeographic diversity patterns regardless of the involved distances.

## Introduction

The Kamchatka Peninsula is located on the northern side of the Kurile–Kamchatka arc and is considered one of the outstanding volcanic regions in the world. The peninsula contains many active volcanoes and numerous related geothermal features including terrestrial geothermal springs, fumaroles, and geysers (Karpov and Naboko, 1990). The Uzon Caldera in Kamchatka is the result of a giant explosion of a stratovolcano during the mid-Pleistocene and the region now contains an array of geothermal features in close proximity (Karpov and Naboko, 1990). A variety of novel thermophilic microorganisms have been isolated from geothermal springs of Kamchatka including *Thermoanaerobacter* taxa. Three of the 14 species presently classified within the *Thermoanaerobacter* genus (May 2013, http://www.bacterio.cict.fr/t/thermoanaerobacter.html) were isolated from hot springs of Kamchatka: *Thermoanaerobacter uzonensis* (Wagner et al., 2008), *Thermoanaerobacter siderophilus* (Slobodkin et al., 1999), and *Thermoanaerobacter sulfurophilus* (Bonch-Osmolovskaya et al., 1997). Furthermore, diverse and unique microbial communities within geothermal springs of the Uzon Caldera have been revealed through 16S rRNA gene clone libraries (Burgess et al., 2011), and high-throughput sequencing of the 16S rRNA gene V6 hypervariable region (D. E. Crowe, pers. communication).

Terrestrial hot springs are frequently regarded as having insular characteristics: they are often well-defined and can be geographically isolated. In addition, geothermal springs in close proximity may have markedly different geochemical properties. For these reasons the comparison of microorganisms from different hot springs provides the opportunity to investigate the spatial patterns of biodiversity. Island-like environments also provide an excellent opportunity to assess gene flow between locations. Understanding characteristics such as genetic variation and gene migration within microbial communities then provides insight into how variations develop and are maintained in natural populations. Biogeographic diversity patterns have been observed for some microorganisms inhabiting terrestrial hot springs, including cyanobacteria (Papke et al., 2003) *Rhodothermus* (Petursdottir et al., 2000), *Thermus* (Hreggvidsson et al., 2006), *Sulfurihydrogenibium* (Takacs-Vesbach et al., 2008), and *Sulfolobus* (Whitaker et al., 2003). Since the 16S rRNA gene sequence is slowly evolving and therefore of little use in intraspecies comparisons (Cooper and Feil, 2004), reports that describe biogeographic patterns within a microbial species have often focused on sequencing and analysis of more rapidly evolving non-coding or protein-coding loci (Whitaker, 2006). In some studies multilocus sequence analysis (MLSA), a technique that utilizes the sequencing of multiple gene fragments to assess the phylogeny and population structure of a group of related strains (Gevers et al., 2005), has been utilized to analyze the spatial diversity patterns of a microbial group (Whitaker et al., 2003; Papke et al., 2007).

Gene flow between sites is significant because of the ensuing potential for recombination within a population. While homologous recombination is reported to occur at varying rates within microbial populations, it has attracted attention because of its importance to fields such as microbial systematics, ecology, population genetics, and evolution (Achtman and Wagner, 2008). As Papke et al. (2007) state, there are potentially two contrasting effects of homologous recombination within a population. Homologous recombination acts as a diversifying force when a pair of strains have strikingly different alleles with one gene, while the remaining genes are identical. Conversely, homologous recombination is a cohesive force when divergent strains share a single identical allele. Considering taxa from terrestrial hot springs, the moderately thermophilic cyanobacterium *Mastigocladus laminosus* was found to be recombining (Miller et al., 2007), as was the population of the aerobic archaeum *Sulfolobus* from the Mutnovsky region of Kamchatka (Whitaker et al., 2005). However, multilocus enzyme electrophoresis of *Rhodothermus marinus* isolates from Iceland indicated that the species is clonal and that recombination occurs rarely (Petursdottir et al., 2000).

Strains of *T. uzonensis*, an obligately anaerobic species within the *Firmicutes* phylum, were repeatedly isolated from geothermal spring samples collected from the Uzon Caldera region of Kamchatka, Far East Russia. The isolation of these microorganisms prompted an initial question of whether spatial patterns of diversity would be observed for this species within this relatively narrow geographical location. To address this question MLSA was performed with 106 strains of *T. uzonensis* from seven pools separated by 140–1600 m within the Uzon Caldera region of Kamchatka, Far East Russia. Because the occurrence and frequency of recombination within and between subpopulation can strongly affect whether biogeographic patterns are observed, we also assessed the influence of homologous recombination on this taxon in this region.

## Materials and methods

### Sample collection and isolation of *Thermoanaerobacter* strains

**Figure 1:**
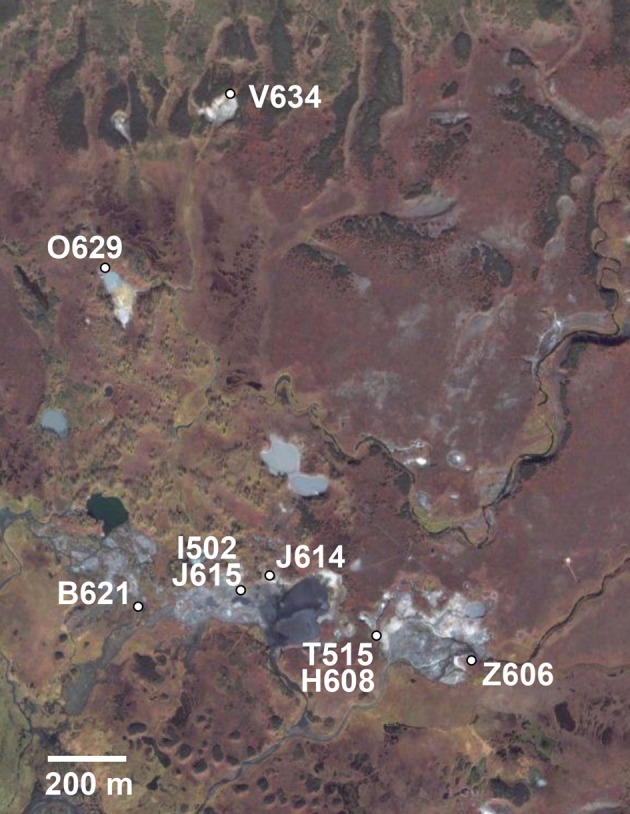
**Location of sample sites in the Uzon Caldera for 106 *T. uzonensis* strains used in this study.** Hot spring positions and abbreviations (Table 1) were overlaid on the satellite image. Image © 2009 Google—Imagery © 2009 DigitalGlobe, GeoEye, Map data © 2009 Geocentre Consulting.

During August 2005 and August 2006, mixed water and sediment samples were collected from geothermal springs within the Uzon Caldera during the Kamchatka Microbial Observatory field seasons (Figure 1). The samples collected had temperature between 49–75°C and pH 5–7.5 (Table 1). Water and sediment samples were transferred to sterilized 100 ml bottles, filled to the brim, sealed with butyl rubber stoppers, transferred to Athens, GA, USA, and stored at 4°C. In the laboratory, 1 ml of mixed water/sediment was transferred to 50 ml Wheaton serum bottles containing 20 ml of an anaerobic mineral medium (Wagner et al., 2008) supplemented with 1 g·l^−1^ glucose, 0.5 g·l^−1^ yeast extract, and 50 mM thiosulfate. Enrichment cultures were incubated at 62°C for 48 h. A 10^−1^ dilution was prepared, streaked onto a 2.15% (w/v) agar plate of the same medium composition, and then incubated anaerobically at 62°C for 48 h. A single colony was selected and re-streaked for isolation on a new agar plate a minimum of two times. Each isolate was derived from its own enrichment culture. To assess culture purity following the repeated single colony isolations, electropherograms of the protein coding loci and 16S rRNA gene were manually examined for correct base calling. Sequences with ambiguous sites were re-sequenced or colonies were isolated anew and loci re-sequenced. Within this study the entire set of isolates was considered the population while the collection of strains derived from a single hot spring was regarded as a subpopulation.

**Table 1:** **Geothermal springs of the Uzon Caldera from which *T. uzonensis* strains were isolated**.

**Geothermal spring (abbreviation)**	**Year**	**Temperature (°C)**	**pH**	**Number of isolates genotyped**
Arkashin Shaft (I502)	2005	72	5	8
Arkashin Shaft (A615)	2006	60–63	5.5	7
Thermophilny (T515)	2005	62–65	7.0–7.5	7
Thermophilny (H608)	2006	53–72	6.0–6.5	19
Burlyashi outflow (B621)	2006	65	7	18
Pulsating Spring (J614)	2006	72	5.2	7
ON1 (O629)	2006	65–72	5.5	12
Vent 1 North (V634)	2006	67	6	18
Zavarzin (Z606)	2006	49–55	5.5	10

Sample sites, sampling year, environmental factors, and number of isolates genotyped.

### PCR amplification of the 16S rRNA gene and protein coding loci

Genomic DNA was isolated with the UltraClean Microbial DNA Isolation kit (Mo Bio). The 16S rRNA gene sequence was amplified with the 27F and 1492R primers (Lane, 1991) using PrimeSTAR HS DNA Polymerase (Takara). The thermal cycler conditions for amplification were: 30 cycles of 98°C for 10 s, 58°C for 5 s, and then 72°C for 90 s. Purification of the amplification product and the subsequent sequencing reaction was performed by Macrogen USA (Rockville, MD).

The universally conserved protein coding genes analyzed in this study were selected from those suggested by Santos and Ochman (2004); *gyrB, lepA, leuS, pyrG, recA, recG, rplB*, and *rpoB*. Primers for the amplification of universally conserved protein coding genes from *Thermoanaerobacter* isolates (Table 2) were designed from the genes of representatives of the family *Thermoanaerobacteracae* with sequenced genomes; *Thermoanaerobacter pseudethanolicus* strain 39E (Refseq: NC_010321), *Caldanaerobacter subterraneus* subsp. *tengcongensis* strain MB4 (Refseq: NC_003869). *Thermoanaerobacter* sp. X514 (Refseq: NC_010320), and *Carboxydothermus hydrogenoformans* Z-2901 (Refseq: NC_007503).

**Table 2:** **Oligonucleotide primers for the amplification of universally conserved protein coding genes from *Thermoanaerobacter uzonensis* isolates**.

**Locus**	**Primer name**	**Oligonucleotide sequence (5′–3′)**
*pyrG*	pyrG-F	AAGYCGCGGCMTATCAGTTGCWRT
	pyrG-R	TGGRTGRAAYTGGGAYGCYACAAA
*leuS*	leuS-F	GYTGYCAAACTGTTCTTGCAAACGARC
	leuS-R	TCATTCTGCTKCCATCAGGKCCCA
*gyrB*	gyrB-F	AGCSGTAAGAAARAGGCCAGGAAT
	gyrB-R	TYCCTCGKAGTGGAAGTATCGCTT
*recA*	recA-F	AGYCARATAGAGAGRCAGTTTGGC
	recA-R	CTCCATAGGAATACCAAGCACCAC
*rplB*	rplB-R	GTGTCTTATARCCYAATGCAGGCT
	rplB-F	ATCTCCCGGCAGACGTCAAAT
*rpoB*	rpoB-R	TCTCTAATGGCTGCWACAACYGGR
	rpoB-F	TACGTCCTGTACAAGTGGGCAACA
*recG*	recG-R	AAATTCTGACCTGCCAACTCTRCC
	recG-F	ACAGGYGYAGTAGARTTAGTSTGG
*lepA*	lepA-R	YTTCCCACCTGTCTCATGCGCTTT
	lepA-F	TTGAGGCGCAAACCCTTGCTAATG

The universally conserved protein coding genes were amplified with Phusion High-Fidelity polymerase PCR Master Mix with HF Buffer (New England Biolabs). Amplification was performed in a Mastercycler ep Gradient thermal cycler (Eppendorf). Conditions for the amplification of the *gyrB, lepA, leuS, pyrG, recG*, and *rpoB* loci were: 98°C for 10 s; then 30 cycles of 98°C for 1 s, 56°C for 5 s, and 72°C for 20 s; and then 72°C for 1 min. Conditions for the amplification of the *recA* and *rplB* loci were: 98°C for 10 s; then 30 cycles of 98°C for 1 s, 56°C for 5 s, and 72°C for 12 s; and then 72°C for 1 min. Purification of the amplification product and the subsequent sequencing reaction was performed by Macrogen USA (Rockville, MD). All nucleotide sequences were deposited to GenBank and are available through the Entrez PopSet database; accession numbers for the different loci from *T. uzonensis* are: 16S rRNA gene, 301133600; *pyrG*, 306992496; *gyrB*, 310780896; *rplB*, 304564022; *recG*, 306992180; *recA*, 304564400; *rpoB*, 304561479; *lepA*, 302120473; and *leuS*, 301133848.

### Analysis of sequence diversity

The 16S rRNA gene sequences were aligned and initially analyzed with Sequencher 4.1 (Gene Codes). Multiple sequence alignments were prepared with NAST (Desantis et al., 2006) through the GreenGenes web application (http://greengenes.lbl.gov/). Multiple sequence alignments of the protein coding gene sequences were prepared with ClustalW (Larkin et al., 2007). Protein-coding loci sequences were initially aligned with the homologous gene sequences from the related *Thermoanaerobacteracae* with sequenced genomes and then checked for spurious insertion or deletions. For every 96-well plate sequenced the locus from one isolate was sequenced multiple times to check that the DNA sequencing was accurate.

Sequence heterogeneity was determined using DnaSP (Rozas and Rozas, 1999) or MEGA 4.1 (Tamura et al., 2007). Characteristics assessed included the total number of polymorphic nucleotide sites, S; the number of alleles for the gene sequence loci, n_a_; and the average number of nucleotide substitutions per site, Pi. Taking into account the deduced primary protein sequence, the number of variable amino acid sites was determined for each locus. Genetic diversity, *H*, was calculated as described by Haubold and Hudson (2000), using the LIAN 3.5 web server (http://adenine.biz.fh-weihenstephan.de/cgi-bin/lian/lian.cgi.pl). This metric was calculated for each protein-coding locus taking into consideration all 106 *T. uzonensis* isolates and for the set of isolates from each hot spring.

A phylogenetic tree based on concatenates of the eight protein coding loci was prepared considering the 49 unique genotypes observed among the set of 106 *T. uzonensis* strains. The phylogenetic analysis was performed in MEGA 5 (Tamura et al., 2011). A concatenated sequence with the same protein coding gene sequences from *Thermoanaerobacter* italicus Ab9^T^ (Hemme et al., 2010) was included in the analysis as an outgroup. Nucleotide substitution models were evaluated and the model having the lowest goodness-of-fit Bayesian Information Criterion value was used to construct a tree using the maximum likelihood method. The initial tree for the maximum likelihood analysis was constructed automatically and the Nearest-Neighbor-Interchange heuristic search method was used to search for topologies that fit the data better. Reliability of the tree topology was assessed with the bootstrap method using 100 replications.

### Calculating *F*_ST_ values and assessing the relationship between divergence and spatial separation

Pairwise *F*_ST_ values between *T. uzonensis* subpopulations from different hot springs were calculated with Arlequin 3.5 (Excoffier and Lischer, 2010), using concatenates of the eight protein-coding loci. *F*_ST_ values were tested for significance against 1000 randomized bootstrap resamplings. The relationship between the genetic divergence, based on nucleotide p-distance from concatenates of the eight protein-coding loci, and spatial separation was examined by calculating Spearman's rho rank correlation value, and the significance level of the Spearman's rho statistic, using the RELATE subprogram within Primer v6 (PRIMER-E Ltd).

### Assessing recombination within the *T. uzonensis* population

For a protein-coding locus, each different allele was assigned a number and the eight-loci sequence type for each isolate was tabulated. The influence of recombination on the population was first assessed by calculating the standardized index of association, *I*^S^_A_, to determine the randomness of the distribution of alleles (Haubold and Hudson, 2000). *I*^S^_A_ values were calculated considering all 106 isolates and considering only the 91 strains isolated from samples collected in 2006. *I*^S^_A_ values were also calculated considering the 49 unique sequence types from 2005 and 2006, as well as the 45 unique genotypes from 2006. Lastly, *I*^S^_A_ values were calculated for each hot spring subpopulation taking into account all isolates and unique genotypes. Recombination in the *T. uzonensis* population was also assessed by examination of single locus variant (SLV) genotypes as described by Feil et al. (2000). Here, SLV genotypes were compiled and the sequence diversity for the variable loci were tabulated. If the variant allele differed by only single nt it was considered a point mutation. The allele was considered to have been the result of homologous recombination if it differed by multiple nt substitutions, or was observed multiple times in the dataset.

## Results

### Isolation of *Thermoanaerobacter* strains

Anaerobic thermophilic strains were isolated from mixed water and sediment samples collected at seven different geothermal springs in the Uzon Caldera. In total, 106 isolates, between seven and 19 from each hot spring sampled, were analyzed by MLSA (Table 1). From 101 strains, the near full-length 16S rRNA gene sequence (≥1337 bp) was obtained and a comparison of the 16S rRNA gene sequence from these isolates revealed ≥98% 16S rRNA gene sequence similarity to each other and to the *Thermoanaerobacter uzonensis* type strain JW/IW010^T^ (Wagner et al., 2008). The geothermal springs from which strains were obtained were separated by at most 1600 m (Figure 1). Each isolate was derived from its own enrichment culture. Hot springs yielding *T. uzonensis* isolates had temperatures of 49–75°C, measured at the location sampled, and circumneutral pH values (Table 1). Attempts to obtain isolates from two additional springs within the Uzon Caldera, “Oil Pool” (75°C, pH 4) and “K4 Well” (60°C; above 100°C in the 16 m deep well shaft, pH 7), were unsuccessful even though 12 or more enrichments where prepared from each sample. The type strain of *T. uzonensis*, JW/IW010^T^, was not included in the MLSA study since it was isolated from a hot spring which at the time of this study had disappeared.

### Protein-coding loci heterogeneity

The protein coding genes used in this study were among those recommended by Santos and Ochman (2004): DNA gyrase subunit B (*gyrB*), GTP-binding protein LepA (*lepA*), leucyl-tRNA synthetase (*leuS*), CTP synthase (*pyrG*), bacterial DNA recombination protein RecA (*recA*), ATP-dependent DNA helicase RecG (*recG*), 50S ribosomal protein L2 (*rplB*), and RNA polymerase subunit B (*rpoB*). The genes are distributed throughout the sequenced genomes of the *Thermoanaerbacteracae* (Hemme et al., 2010; detailed data not shown). To minimize the inclusion of apparent sequence heterogeneity due to DNA sequencing errors the protein-coding loci were amplified with Phusion High-Fidelity DNA Polymerase in HF Buffer (New England BioLabs, Inc).

There were 148 variable sites from a total of 8003 bp in common across the eight protein-coding loci. All loci were polymorphic, however, the amount of variation at each locus differed (Table 3). For example, the number of variable nucleotide sites (S) observed for a locus varied from 3 for the *rplB* locus, to 42 for the *recG* locus. The deduced primary protein sequence revealed that most nucleotide substitutions were synonymous (Table 3).

**Table 3:** **Characteristics of eight protein coding loci examined from the set of 106 *T. uzonensis* strains isolated from hot springs of the Uzon Caldera**.

	***gyrB***	***lepA***	***leuS***	***pyrG***	***recA***	***recG***	***rplB***	***rpoB***
Length (nt)	1111	1264	1040	1204	739	1227	507	911
Number of alleles (*n*_a_)	4	13	10	25	7	16	4	8
Genetic diversity, *H*	0.32	0.62	0.75	0.93	0.36	0.89	0.49	0.57
Variable nt sites (S)	16	14	24	30	6	42	3	13
Average nucleotide diversity (Pi) per 100 nt sites	0.185	0.095	0.269	0.654	0.052	0.95	0.11	0.363
Variable amino acid residues	8	4	13	12	2	22	1	3

Characteristics calculated for each protein coding locus were: nucleotide sequence length, nt; number of alleles, n_a_; total number of polymorphic nucleotide sites, S; average number of nucleotide substitutions per site, Pi; and the variable amino acid residues.

### Genetic differentiation of *T. uzonensis* subpopulations

Genetic differentiation of the subpopulations from different hot spring was assessed by calculation of the pairwise *F*_ST_ values (Table 4). The *F*_ST_ values ranged from 0.082 to 0.706, and most values were found to be significant based on a bootstrap resampling test. Two of the five comparisons that were found to not be significantly different were the comparisons between Arkashin over multiple years and Thermophilny over multiple years.

**Table 4:** ***T. uzonensis* hot spring subpopulation pairwise *F*_ST_ values**.

	**A615**	**I502**	**B621**	**H608**	**T515**	**J614**	**O629**	**V634**
**I502**	0.347^*^							
**B621**	0.175	0.521						
**H608**	0.374	0.657	0.147					
**T515**	0.287	0.706	0.082^*^	0.093^*^				
**J614**	0.301	0.643	0.280	0.441	0.268^*^			
**O629**	0.336	0.619	0.198	0.331	0.304	0.359		
**V634**	0.430	0.698	0.182	0.381	0.333	0.426	0.214	
**Z606**	0.238^*^	0.565	0.217	0.407	0.311	0.364	0.367	0.382

Hot spring abbreviations given in Table 1. F_ST_ values were tested for significance against 1000 randomized bootstrap resamplings;

* indicates P > 0.01.

Hot springs from which *T. uzonensis* isolates were derived were separated by distances that varied from about 140–1600 m (Figure 1), measured using a QuickBird (DigitalGlobe) satellite image (D. E. Crowe, personal communication). The relationship between the spatial separation of the hot springs and the genetic divergence of the *T. uzonensis* isolates was assessed by calculation of the Spearman's rank correlation coefficient: rho = 0.086, significance level of sample statistic: 0.83%.

### Distribution of alleles and genotypes

**Figure 2:**
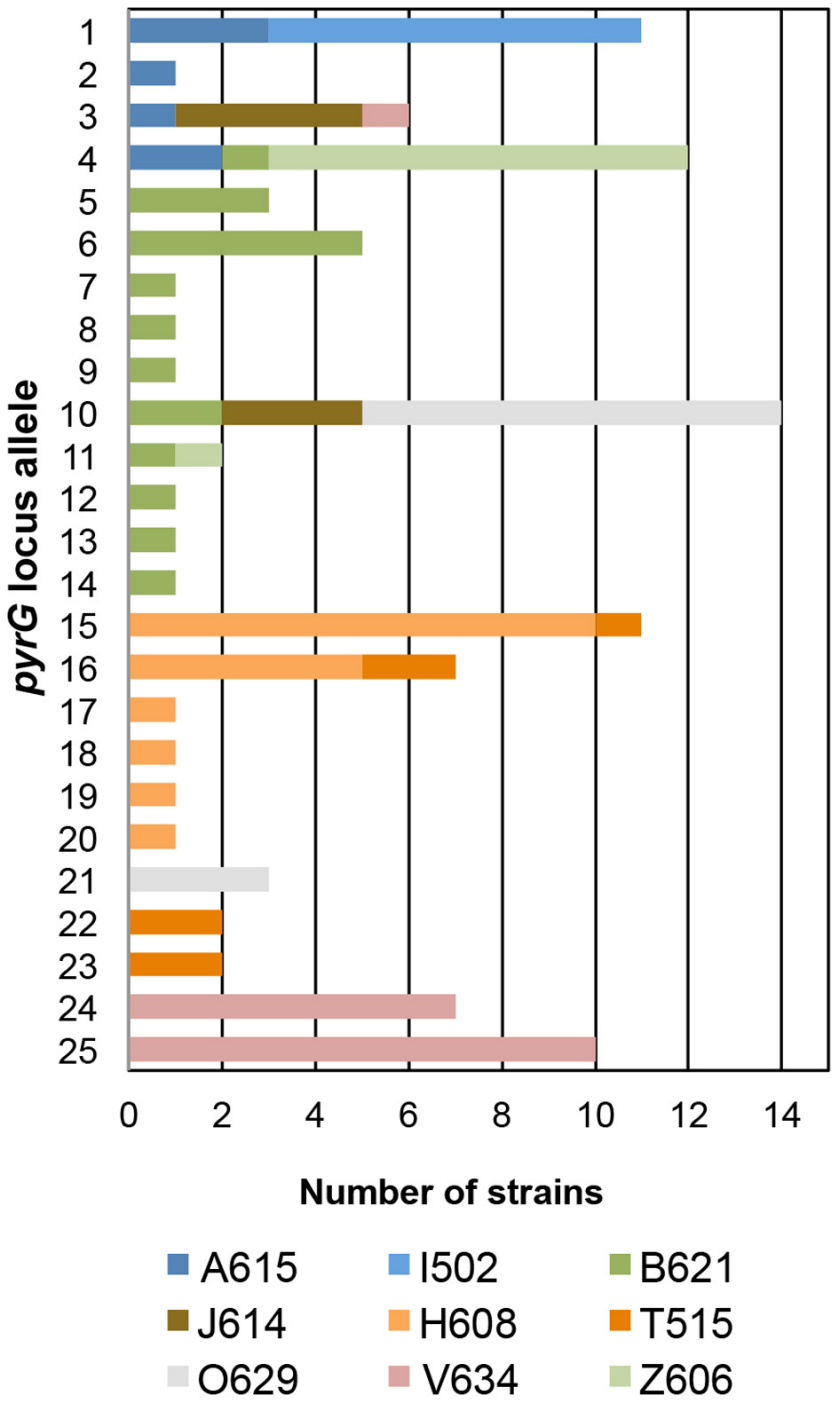
**Distribution of *pyrG* alleles among *T. uzonensis* isolates.** Bars are color coded and correspond to the hot spring from which the *T. uzonensis* isolates were derived. Geothermal spring abbreviations are given in Table 1.

In the *T. uzonensis* population, the number of alleles at a particular protein-coding locus varied from 4 (*rplB* and *gyrB*) to 25 (*pyrG*). The genetic diversity, *H*, varied from 0.32 for *gyrB* to 0.93 for *pyrG* for the individual protein-coding loci (Table 3) and the average was 0.62. Correspondently, some alleles were found in a high proportion of the *T. uzonensis* population, e.g., *gyrB* allele 1, 82.1%; *recA* allele 1, 79.2%; and *rplB* allele 3, 67.9% (detailed data not shown). The distribution of alleles within a hot spring subpopulation was also examined. The seven isolates from Arkashin Shaft 2006 shared the same *gyrB, lepA*, and *recA* allele, but had comparatively high variation at the *leuS, pyrG*, and *recG* loci (Table 5). Among the 18 *T. uzonensis* isolates from Thermophilny 2006, there was a single *gyrB* locus allele, while the other seven loci were variable. All of the isolates from Arkashin Shaft 2005 had the same protein-coding loci sequence type, whereas considerable variation was found at all loci from the set of the Burlyashi outflow isolates (Table 5). Occasionally a particular allele was only observed within the *T. uzonensis* subpopulation from one hot spring and this was especially evident at the *pyrG* locus (Figure 2). For example, *pyrG* alleles 24 and 25 were only found in isolates from Vent 1 North (closest spring analyzed was ON1, approximately 530 m away), *pyrG* alleles 15 and 16 were only in strains from Thermophilny isolated in 2005 and 2006 (closest spring examined was Zavarzin, about 210 m distant), and *pyrG* allele 1 was only found in *T. uzonensis* strains from Arkashin in both 2005 and 2006 (closest spring analyzed, Pulsating Spring about 140 m away).

**Table 5:** **Characteristics of protein coding loci examined from *T. uzonensis* subpopulations from different hot springs in the Uzon Caldera**.

**Hot Spring (year)**		***gyrB***	***lepA***	***leuS***	***pyrG***	***recA***	***recG***	***rplB***	***rpoB***
Arkashin Shaft (2006)	*n*_a_	1	1	4	4	1	3	3	2
	*H*	0	0	0.71	0.81	0	0.71	0.67	0.48
	S	0	0	16	16	0	19	2	3
Burlyashi Outflow (2006)	*n*_a_	3	4	6	11	4	6	3	5
	*H*	0.52	0.40	0.76	0.91	0.54	0.76	0.31	0.48
	S	4	4	22	22	3	33	2	11
Thermophilny (2006)	*n*_a_	1	3	5	6	3	6	3	2
	*H*	0	0.20	0.65	0.68	0.57	0.68	0.11	0.20
	S	0	3	15	22	2	16	2	5
Thermophilny (2005)	*n*_a_	2	4	4	4	3	4	1	1
	*H*	0.48	0.71	0.86	0.86	0.52	0.86	0	0
	S	12	8	3	17	2	9	0	0
Pulsating Spring (2006)	*n*_a_	2	3	2	2	1	2	2	1
	*H*	0.57	0.71	0.57	0.57	0	0.57	0.57	0
	S	15	2	1	6	0	13	1	0
ON1 (2006)	*n*_a_	2	4	2	2	1	4	3	4
	*H*	0.17	0.77	0.41	0.41	0	0.56	0.53	0.64
	S	3	3	1	17	0	24	2	10
Vent 1 North (2006)	*n*_a_	2	5	3	3	1	3	2	2
	*H*	0.11	0.71	0.57	0.57	0	0.57	0.11	0.11
	S	12	4	2	8	0	28	1	5
Zavarzin (2006)	*n*_a_	2	3	3	2	2	4	2	3
	*H*	0.36	0.60	0.60	0.20	0.47	0.71	0.47	0.62
	S	3	2	2	8	1	20	1	9

The eight isolates from Arkashin 2005 had the same genotype. Characteristics calculated for each protein coding locus were: number of alleles, n_a_; genetic diversity, H; and number of polymorphic nucleotide sites, S.

**Figure 3:**
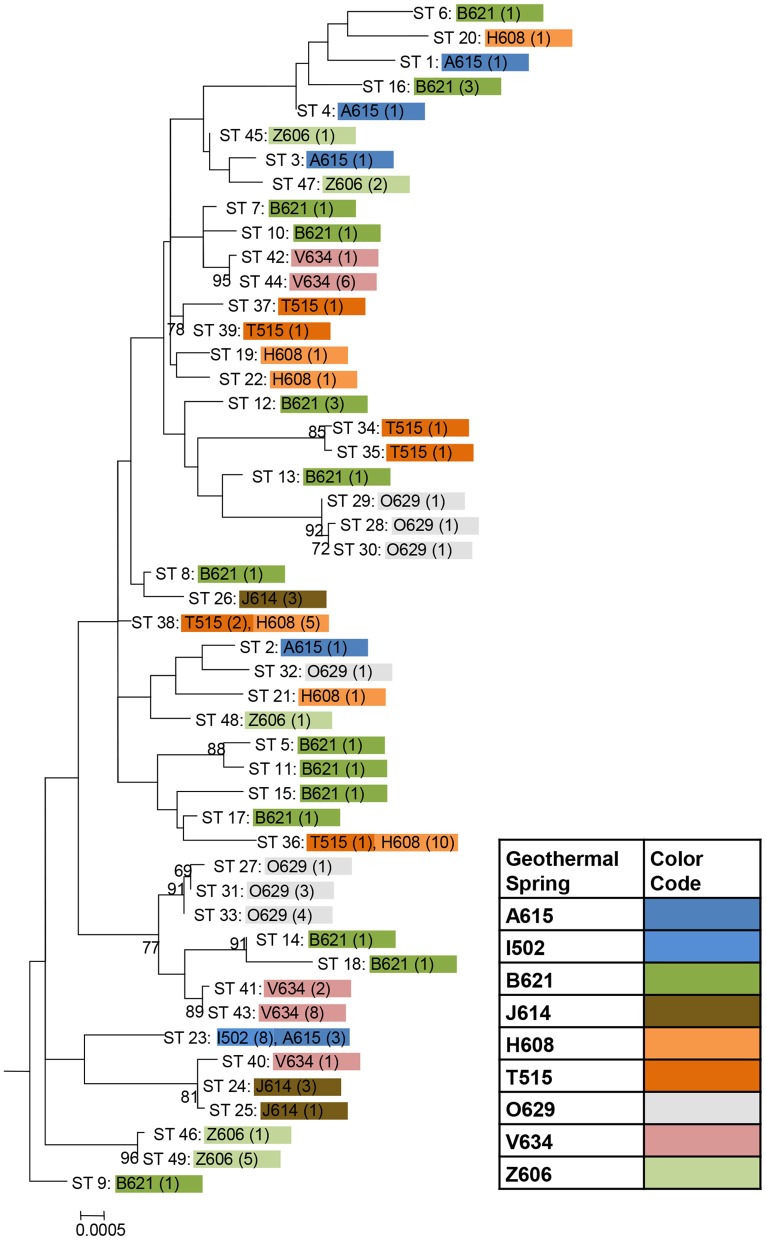
**Phylogenetic tree based on concatenates of the eight protein coding loci from the 49 unique sequence types among 106 *T. uzonensis* strains.** ST designations match those given in Table 6 and are color coded according to hot spring origin. The number of strains having the particular ST is given in parentheses. Hot spring abbreviations are given in Table 1. The maximum likelihood tree was constructed using the Hasegawa-Kishino-Yano model with a rates among sites setting of gamma distributed with invariant sites. Only bootstrap proportions of 50 or higher are included on the tree. The tree is drawn to scale, with branch lengths measured in the number of substitutions per site.

Among the 106 *T. uzonensis* strains there were 49 unique sequence types (STs, Table 6). A majority of the STs, 35 of the 49, were unique to a single isolate and at most a sequence type was held by 11 isolates (STs 23 and 36; Table 6). Within the Uzon Caldera, isolates with identical genotypes were, in all instances, derived from the same hot springs. *T. uzonensis* isolates were obtained from samples collected at the Arkashin and Thermophilny springs in 2005 and 2006 and for both springs, strains with the same allelic profile were obtained over the 2 years. There were 11 pairs of SLVs among the 49 genotypes. Of these SLVs pairs, 10 were of genotypes held by isolates from the same hot spring (Table 7). The phylogenetic tree inferred from concatenates of the protein coding loci showed that genotypes of strains isolated from the same geothermal spring occasionally clustered together (Figure 3). Most bootstrap values were below 50%, which indicated minimal reliability in the tree topology.

**Table 6:** **Summary of *T. uzonensis* sequence types**.

**Sequence type**	**Allelic profile**	**No. of isolates**	**Spring**
1	1;1;2;2;1;2;2;1	1	Arkashin 2006
2	1;1;1;3;1;3;3;2	1	Arkashin 2006
3	1;1;3;4;1;2;1;1	1	Arkashin 2006
4	1;1;4;4;1;2;3;2	1	Arkashin 2006
23	1;1;1;1;1;1;1;1	11	Arkashin 2005, 2006
24	4;1;9;3;1;10;2;2	3	Pulsating Spring 2006
25	4;7;9;3;1;10;2;2	1	Pulsating Spring 2006
26	3;6;5;10;1;2;3;5	3	Pulsating Spring 2006
27	1;8;5;10;1;11;1;7	1	ON1 2006
28	1;7;1;21;1;12;3;8	1	ON1 2006
29	1;1;1;21;1;9;3;8	1	ON1 2006
30	1;7;1;21;1;9;3;8	1	ON1 2006
31	1;8;5;10;1;11;1;3	3	ON1 2006
32	3;7;5;10;1;3;2;2	1	ON1 2006
33	1;4;5;10;1;11;1;3	4	ON1 2006
40	4;4;9;3;1;6;2;2	1	Vent 1 North 2006
41	1;12;1;25;1;11;3;3	2	Vent 1 North 2006
42	1;13;5;24;1;3;3;3	1	Vent 1 North 2006
43	1;11;1;25;1;11;3;3	8	Vent 1 North 2006
44	1;1;5;24;1;3;3;3	6	Vent 1 North 2006
45	1;1;5;4;7;2;3;2	1	Zavarzin 2006
46	1;4;1;4;1;15;3;3	1	Zavarzin 2006
47	3;1;5;4;7;2;2;6	2	Zavarzin 2006
48	1;11;9;11;1;16;2;2	1	Zavarzin 2006
49	1;4;1;4;1;14;3;3	5	Zavarzin 2006
5	1;1;5;6;1;4;2;4	1	Burylashi 2006
6	1;1;6;7;3;5;4;2	1	Burylashi 2006
7	1;1;1;8;1;3;3;3	1	Burylashi 2006
8	1;1;5;9;1;2;3;3	1	Burylashi 2006
9	3;2;5;10;1;6;3;5	1	Burylashi 2006
10	1;1;5;4;1;3;3;2	1	Burylashi 2006
11	1;3;5;11;1;4;2;6	1	Burylashi 2006
12	1;1;5;5;2;2;3;3	3	Burylashi 2006
13	3;1;1;12;1;2;3;3	1	Burylashi 2006
14	1;4;1;13;1;7;3;3	1	Burylashi 2006
15	2;1;7;10;4;4;3;3	1	Burylashi 2006
16	2;1;4;6;1;2;3;3	3	Burylashi 2006
17	1;1;8;6;4;4;3;3	1	Burylashi 2006
18	1;4;1;14;1;7;3;3	1	Burylashi 2006
19	1;5;9;17;1;8;3;3	1	Thermophilny 2006
20	1;1;10;18;1;3;2;2	1	Thermophilny 2006
21	1;1;1;19;5;9;3;2	1	Thermophilny 2006
22	1;4;5;20;1;2;3;3	1	Thermophilny 2006
34	4;1;1;22;6;13;3;3	1	Thermophilny 2005
35	4;7;1;22;1;13;3;3	1	Thermophilny 2005
36	1;1;8;15;4;4;3;3	11	Thermophilny 2005, 2006
37	1;9;5;23;1;2;3;3	1	Thermophilny 2005
38	1;1;9;16;1;5;3;3	7	Thermophilny 2005, 2006
39	1;10;5;23;1;2;3;3	1	Thermophilny 2005

Allelic profile protein-coding loci order: gyrB, lepA, leuS, pyrG, recA, recG, rplB, and rpoB.

**Table 7:** **Examination of single locus variants among the *T. uzonesis* population**.

**Sequence type 1**		**Sequence type 2**		**Locus, bp differences**	**SLV analysis**
**ST**	**Spring, year**	**ST**	**Spring, year**		
14	Burylashi, 2006	18	Burylashi, 2006	*pyrG*, 10	R
17	Burylashi, 2006	36	Thermophilny, 2005, 2006	*pyrG*, 13	R
24	Pulsating Spring, 2006	25	Pulsating Spring, 2006	*lepA*, 1	R
27	ON1, 2006	31	ON1, 2006	*rpoB*, 2	R
28	ON1, 2006	30	ON1, 2006	*recG*, 1	M
29	ON1, 2006	30	ON1, 2006	*lepA*, 1	R
31	ON1, 2006	33	ON1, 2006	*lepA*, 1	R
37	Thermophilny, 2005	39	Thermophilny, 2005	*lepA*, 6	R
41	Vent 1 North, 2006	43	Vent 1 North, 2006	*lepA*, 1	M
42	Vent 1 North, 2006	44	Vent 1 North, 2006	*lepA*, 1	M
46	Zavarzin, 2006	49	Zavarzin, 2006	*recG*, 1	M

Each row lists the SLV sequence types, the differing protein coding locus and number of polymorphic nucleotide sites, and whether the locus was deemed to be the result of recombination, R; or mutation, M, according to the SLV analysis.

### Assessing the influence of recombination on the *T. uzonensis* population of the uzon caldera

The influence of recombination on the *T. uzonensis* population structure was assessed by calculating the standardized index of association, *I*^S^_A_, to determine the randomness of the distribution of alleles (Haubold and Hudson, 2000). This statistic is expected to be zero in populations that are freely recombining and greater than zero if there is linkage disequilibrium. *I*^S^_A_ was estimated to be 0.086 when all 106 isolates were analyzed and this value was significantly different from zero (P < 0.001). However, when *I*^S^_A_ is calculated using only the 49 unique STs the value decreases to 0.028 (*P* = 0.028). Similar values were obtained when the analysis was restricted to the strains isolated in 2006 (Table 8). The *I*^S^_A_ statistic was also calculated for the set of isolates from each spring separately and the *I*^S^_A_ values were higher when the calculation was restricted to the subpopulations (Table 8).

**Table 8:** **Standardized index of association values calculated with the *T. uzonensis* MLSA dataset**.

**Set of *T. uzonensis* strains analyzed**	**All isolates**	**Unique genotypes**
Uzon Caldera isolates from 2005 and 2006	106 isolates *I*^S^_A_ = 0.086 (*P* < 0.001)	49 ST *I*_AS_ = 0.028 (*P* = 0.028)
Uzon Caldera isolates from 2006	91 isolates *I*^S^_A_ = 0.090 (*P* < 0.001)	45 ST *I*^S^_A_ = 0.030 (*P* = 0.021)
Arkashin Shaft 2006	7 isolates *I*^S^_A_ = 0.209 (*P* < 0.001)	4 ST *I*^S^_A_ = 0.041 (*P* = 0.286)
Burlyashi outflow (2006)	18 isolates *I*^S^_A_ = 0.071 (*P* = 0.012)	14 ST *I*^S^_A_ = 0.018 (*P* = 0.293)
Thermophilny (2005)	7 isolates *I*^S^_A_ = 0.179 (*P* < 0.001)	6 ST *I*^S^_A_ = 0.141 (*P* = 0.028)
Thermophilny (2006)	19 isolates *I*^S^_A_ = 0.390 (*P* < 0.001)	4 ST *I*^S^_A_ = −0.027 (*P* = 0.537)
Pulsating Spring (2006)	7 isolates *I*^S^_A_ = 0.841 (*P* < 0.001)	3 ST NA
ON1 (2006)	12 isolates *I*^S^_A_ = 0.463 (*P* < 0.001)	7 ST *I*^S^_A_ = 0.346 (*P* < 0.001)
Vent 1 North (2006)	18 isolates *I*^S^_A_ = 0.478 (*P* < 0.001)	5 ST *I*^S^_A_ = 0.524 (*P* < 0.001)
Zavarzin (2006)	10 isolates *I*^S^_A_ = 0.545 (*P* < 0.001)	5 ST *I*^S^_A_ = 0.211 (*P* = 0.007)

As stated above, there were 11 SLVs pairs among the *T. uzonensis* genotypes. Following the method of binning recombination and mutation events (Feil et al., 2000), SLVs are considered to be the result of mutation if they are single nucleotide changes and are unique in the dataset, whereas recombination events can have single or multiple nucleotide changes and are encountered several times independently. Of the 11 SLVs, seven appear to be due to recombination events while four are due to mutation (Table 7).

## Discussion

The study of variability in natural populations is important because it can provide insight into the evolutionary forces through which variation develops and is maintained (Smith, 1995). The diversity of 106 *T. uzonensis* strains, isolated from seven hot springs within one region, was assessed through the sequencing and analysis of eight protein coding loci. This MLSA revealed that while recombination occurs, the subpopulations from different springs in this region are genetically differentiated. The results presented here are based on an initial culture-dependent step where the focus was to obtain similar strains under identical isolation conditions. As such, we acknowledge that this set of *T. uzonensis* strains may not necessarily reflect the full diversity of *T. uzonensis* in this environment.

A 16S rRNA gene sequence similarity of ≥97% between strains is evidence that the isolates may belong within the same species (Stackebrandt and Goebel, 1994). Therefore the high (≥98%) 16S rRNA gene sequence similarity to each other and to *Thermoanaerobacter uzonensis* strain JW/IW010^T^ (Wagner et al., 2008) supported the view that these isolates belong to the same species. This idea was further bolstered by the MLSA results, in particular the relatively low amount of nucleotide sequence variation at the protein coding loci.

The eight protein coding loci examined within the *T. uzonensis* population were polymorphic and a range of variation was observed across the different loci (Table 3). Comparable levels of sequence diversity have been observed in other MLSA-based studies of the population structure of a microbial species within a region. The number of polymorphic sites per locus varied from 2 to 12 for six protein-coding loci from 60 *Sulfolobus* isolates from the Mutnovsky region of Kamchatka, Far East Russia (Whitaker et al., 2005), and among 36 *Halorubrum* isolates from two solar salterns at Santa Pola near Alicante, Spain, four protein-coding loci had 30–61 polymorphic sites per locus (Papke et al., 2004).

The spatial scale of microbial diversity studies are important to consider. Previous authors have noted that environmental factors or historical contingencies are thought to influence patterns of genetic variation on smaller scales, while isolation distance is believed to supersede environmental effects at intercontinental scales (Takacs-Vesbach et al., 2008). For example, greater divergence among the protein-coding loci was reported for both *Sulfolobus* (Whitaker et al., 2003) and *Halorubrum* (Papke et al., 2007) when the isolates analyzed were from regions separated by ≥250 km. While the focus of this report is the diversity of *T. uzonensis* within Uzon Caldera hot springs, similar strains were also isolated from two hot springs within the Geyser Valley region, 10 km east of the Uzon Caldera, and one hot spring from the Mutnovsky volcano region, located 250 km south of the Uzon Caldera and Geyser Valley. Analyses with the protein-coding loci from these strains revealed, with few exceptions, an increase in genetic divergence with an increase in geographic distance (data not shown).

The genetic diversity values, *H*, calculated for the *gyrB, recA* and *rplB* loci were relatively low (Table 3), and for these three genes a particular allele was found held by a high percentage of the *T. uzonensis* strains. A similar observation was made for a set of *Halorubrum* isolates where the a single *bop* allele was found in >85% of the strains and this was interpreted as being in part the result of selection, which drove the allele to high frequency (Papke, 2009). This explanation is compatible with some of the genes examined within the *T. uzonensis* population. The most notable exceptions were the *pyrG* and *recG* loci. Balancing selection may, in part, explain the diverse set of *recG* alleles observed within the population. Interestingly, for the *pyrG* locus a particular allele was often only found among the strains from a single hot spring (Figure 2). This could be the result of genetic drift within subpopulations, a neutral force, or positive selection of the particular allele within the hot spring subpopulation. One potential observation from a MLSA study would be the clustering of genotypes according to origin in a phylogenetic tree prepared from concatenates of the different loci. Only limited clustering was sequence types was observed (Figure 3), but this was not an unexpected result. The genes included in this study may have been influenced by different evolutionary processes, which potentially complicates phylogenetic analyses, and moreover there was evidence for homologous recombination in this population.

The investigated hot springs were separated by distances of 140–1600 m (Figure 1), and therefore *T. uzonensis* strains that developed in one pool could be distributed among the springs of the Uzon Caldera by wind, water, and local fauna (e.g., birds and brown bears). Moreover, there was evidence that gene flow between regions occurs as the same *rplB* allele was found in isolates from the Uzon Caldera, Geyser Valley, and Mutnovsky volcano regions (data not shown). Many of the described *Thermoanaerobacter* taxa, including *T. uzonensis* JW/IW010^T^, are known to form spores or contain sporulation-specific genes (Brill and Wiegel, 1997; Onyenwoke et al., 2004; Wagner et al., 2008). Sporulation would undoubtedly contribute to the ability of *T. uzonensis* to survive transport between geothermal springs within and between regions, a form of passive dispersal as discussed by Martiny et al. (2006). Despite the close spatial proximity of the hot springs in this study, the pairwise *F*_ST_ values indicated that there was a small but significant level of genetic differentiation between most subpopulations (Table 4). There was a negligible association between the genetic divergence of *T. uzonensis* isolates and the geographic separation of the corresponding hot springs. This observation supports the concept mentioned earlier: that on smaller scales, as mainly investigated in this study, environmental factors or historical contingencies are believed to be of primary importance in determining whether patterns of genetic variation exist (Takacs-Vesbach et al., 2008).

Although the different geothermal springs sampled had approximately the same temperature and pH where the sample was collected (Table 1), there were other physicochemical differences. For example, the Arkashin Shaft spring is geochemically distinct in that it has a high arsenic concentration (4252 mg kg^−1^ measured within Arkashin Shaft in 2006; Burgess et al., 2011). The hot springs in this study also differed in size and physical setting. Previous studies have revealed that at the community level microbial richness increases with habitat volume (Bell et al., 2005; Van Der Gast et al., 2005). The Burlyashi spring was the largest hot spring sampled (personal observation) and this property may, in part, explain the high diversity observed among the 18 strains from the Burlyashi spring outflow (Table 5).

Analyses of microbial populations have revealed that while homologous recombination occurs at widely varying rates, it has been observed among most taxa (Papke et al., 2007). The genomes of *Thermoanaerobacter* strains isolated from the Piceance Basin, Colorado, USA, revealed considerable recombination (C. L. Hemme, unpublished results). Our results show that the *T. uzonensis* population in the Uzon Caldera was influenced by frequent recombination. However, the difference in *I*^S^_A_ values calculated from all 106 isolates and the 49 unique STs (Table 8) provides evidence of an “epidemic” population structure, in which recombination occurs while particular clones also rise in frequency. *Sulfolobus* isolates from two hot springs in the Mutnovsky region of Kamchatka, similarly had an epidemic population structure (Whitaker et al., 2005), and this population structure has been proposed as indicating that certain clonal types may have increased fitness. Within the *T. uzonensis* population the view that a sequence type held by multiple isolates has increased fitness is particularly intriguing considering the sequence types found over consecutive years within the Arkashin and Thermophilny springs.

While there is great potential for *T. uzonensis* strains to be transferred between hot springs within the Uzon Caldera, isolates with identical sequence types were always derived from the same spring and SLV sequence types were usually isolated from a single site. This observation, along with the pairwise *F*_ST_ values, suggests that the *T. uzonensis* subpopulations within different hot springs are ecologically distinct and future studies could be performed to further examine the genetic and physiological differences between strains. Moreover, the genetic differentiation of subpopulations is likely influenced by the physicochemical differences between the geothermal springs. While there was strong evidence for frequent recombination within the *T. uzonensis* population, the observation that subpopulations were genetically differentiated is not unexpected. Simulations performed by Hanage et al. (2006) demonstrated that distinct clusters of similar genotypes can emerge in populations with a range of mutation and recombination rates. This MLSA additionally suggests that there are interesting genome dynamics within the *T. uzonensis* taxon with some alleles approaching fixation throughout the entire population. Other alleles were only seen within particular subpopulations, potentially the result of positive selection within the hot spring or genetic drift. Comparing the genomes of strains from different springs would provide insight into the genomic context of the protein-coding loci herein examined and would provide information concerning the variation in gene content among strains. While physical isolation of subpopulations is an important factor that influences the genetic divergence between sites, this work shows that differentiated populations can emerge within a region.

### Conflict of interest statement

The authors declare that the research was conducted in the absence of any commercial or financial relationships that could be construed as a potential conflict of interest.
