# Supplementary material for: Multilocus sequence analysis of Thermoanaerobacter isolates reveals recombining, but differentiated, populations from geothermal springs of the Uzon Caldera, Kamchatka, Russia
Source: Front Microbiol. 2013 Jun 21;4:169. doi: 10.3389/fmicb.2013.00169 (PMC3689144; doi:10.3389/fmicb.2013.00169)
Supplement: Supplementary file 1 [file DataSheet1.ZIP › MAY13_S_info/18MAY2013_TBIOGEO_FM_supplementalTables.docx]

| **Table S1. Sequence type and allelic profile data for *T. uzonensis* strains analyzed by MLSA** | | | | |
| --- | --- | --- | --- | --- |
| **Sequence type** | **Allelic profile** | **No. of isolates** | **Spring** | ***T. uzonensis* strains with the allelic profile** |
| 1 | 1;1;2;2;1;2;2;1 | 1 | Arkashin 2006 | A615_35 |
| 2 | 1;1;1;3;1;3;3;2 | 1 | Arkashin 2006 | A615_37 |
| 3 | 1;1;3;4;1;2;1;1 | 1 | Arkashin 2006 | A615_39 |
| 4 | 1;1;4;4;1;2;3;2 | 1 | Arkashin 2006 | A615_65 |
| 5 | 1;1;5;6;1;4;2;4 | 1 | Burylashi 2006 | B621_1 |
| 6 | 1;1;6;7;3;5;4;2 | 1 | Burylashi 2006 | B621_2 |
| 7 | 1;1;1;8;1;3;3;3 | 1 | Burylashi 2006 | B621_3 |
| 8 | 1;1;5;9;1;2;3;3 | 1 | Burylashi 2006 | B621_4 |
| 9 | 3;2;5;10;1;6;3;5 | 1 | Burylashi 2006 | B621_72 |
| 10 | 1;1;5;4;1;3;3;2 | 1 | Burylashi 2006 | B621_74 |
| 11 | 1;3;5;11;1;4;2;6 | 1 | Burylashi 2006 | B621_77 |
| 12 | 1;1;5;5;2;2;3;3 | 3 | Burylashi 2006 | B621_78, B621_10, B621_6 |
| 13 | 3;1;1;12;1;2;3;3 | 1 | Burylashi 2006 | B621_80 |
| 14 | 1;4;1;13;1;7;3;3 | 1 | Burylashi 2006 | B621_83 |
| 15 | 2;1;7;10;4;4;3;3 | 1 | Burylashi 2006 | B621_87 |
| 16 | 2;1;4;6;1;2;3;3 | 3 | Burylashi 2006 | B621_90, B621_71, B621_89_1 |
| 17 | 1;1;8;6;4;4;3;3 | 1 | Burylashi 2006 | B621_93 |
| 18 | 1;4;1;14;1;7;3;3 | 1 | Burylashi 2006 | B621_94 |
| 19 | 1;5;9;17;1;8;3;3 | 1 | Thermophilny 2006 | H608_78 |
| 20 | 1;1;10;18;1;3;2;2 | 1 | Thermophilny 2006 | H608_79 |
| 21 | 1;1;1;19;5;9;3;2 | 1 | Thermophilny 2006 | H608_81 |
| 22 | 1;4;5;20;1;2;3;3 | 1 | Thermophilny 2006 | H608_93 |
| 23 | 1;1;1;1;1;1;1;1 | 11 | Arkashin 2005, 2006 | I502_49, A615_31, A615_32, A615_62, I502_40, I502_41, I502_42, I502_43, I502_45, I502_46, I502_48 |
| 24 | 4;1;9;3;1;10;2;2 | 3 | Pulsating Spring 2006 | J614_63, J614_60, J614_61 |
| 25 | 4;7;9;3;1;10;2;2 | 1 | Pulsating Spring 2006 | J614_65 |
| 26 | 3;6;5;10;1;2;3;5 | 3 | Pulsating Spring 2006 | J614_9, J614_62_1, J614_7 |
| 27 | 1;8;5;10;1;11;1;7 | 1 | ON1 2006 | O629_40 |
| 28 | 1;7;1;21;1;12;3;8 | 1 | ON1 2006 | O629_42 |
| **Table S1 (continued). Sequence type and allelic profile data for *T. uzonensis* strains analyzed by MLSA** | | | | |
| **Sequence type** | **Allelic profile** | **No. of isolates** | **Spring** | ***T. uzonensis* strains with the allelic profile** |
| 29 | 1;1;1;21;1;9;3;8 | 1 | ON1 2006 | O629_47 |
| 30 | 1;7;1;21;1;9;3;8 | 1 | ON1 2006 | O629_48 |
| 31 | 1;8;5;10;1;11;1;3 | 3 | ON1 2006 | O629_50, O629_43, O629_49 |
| 32 | 3;7;5;10;1;3;2;2 | 1 | ON1 2006 | O629_51 |
| 33 | 1;4;5;10;1;11;1;3 | 4 | ON1 2006 | O629_7, O629_44, O629_45, O629_6 |
| 34 | 4;1;1;22;6;13;3;3 | 1 | Thermophilny 2005 | T515_1 |
| 35 | 4;7;1;22;1;13;3;3 | 1 | Thermophilny 2005 | T515_3 |
| 36 | 1;1;8;15;4;4;3;3 | 11 | Thermophilny 2005, 2006 | T515_40, H608_1, H608_10, H608_2, H608_41, H608_6, H608_71, H608_72, H608_84, H608_85, H608_88 |
| 37 | 1;9;5;23;1;2;3;3 | 1 | Thermophilny 2005 | T515_41 |
| 38 | 1;1;9;16;1;5;3;3 | 7 | Thermophilny 2005, 2006 | T515_42, H608_3, H608_4, H608_42, H608_77, H608_91, T515_10 |
| 39 | 1;10;5;23;1;2;3;3 | 1 | Thermophilny 2005 | T515_44 |
| 40 | 4;4;9;3;1;6;2;2 | 1 | Vent 1 North 2006 | V634_10 |
| 41 | 1;12;1;25;1;11;3;3 | 2 | Vent 1 North 2006 | V634_82, V634_5 |
| 42 | 1;13;5;24;1;3;3;3 | 1 | Vent 1 North 2006 | V634_83 |
| 43 | 1;11;1;25;1;11;3;3 | 8 | Vent 1 North 2006 | V634_84, V634_3, V634_71, V634_73, V634_74, V634_77, V634_8, V634_81 |
| 44 | 1;1;5;24;1;3;3;3 | 6 | Vent 1 North 2006 | V634_9, V634_1, V634_2, V634_6, V634_7, V634_85 |
| 45 | 1;1;5;4;7;2;3;2 | 1 | Zavarzin 2006 | Z606_36 |
| 46 | 1;4;1;4;1;15;3;3 | 1 | Zavarzin 2006 | Z606_70 |
| 47 | 3;1;5;4;7;2;2;6 | 2 | Zavarzin 2006 | Z606_73, Z606_72 |
| 48 | 1;11;9;11;1;16;2;2 | 1 | Zavarzin 2006 | Z606_81 |
| 49 | 1;4;1;4;1;14;3;3 | 5 | Zavarzin 2006 | Z606_9, Z606_3, Z606_38, Z606_71, Z606_77 |

| **Table S2. GenBank accession numbers for the gene sequences of *T. uzonensis* strains analyzed by MLSA** | | | | | | | | | |
| --- | --- | --- | --- | --- | --- | --- | --- | --- | --- |
| ***T. uzonensis* strain** | ***gyrB*** | ***rplB*** | ***pyrG*** | ***recG*** | ***recA*** | ***rpoB*** | ***lepA*** | ***leuS*** | **16S rRNA** |
| A615_31 | HM182416 | HM190570 | HM191136 | HM190780 | HM190930 | HM190334 | HM182673 | HM182834 | HM182372 |
| A615_32 | HM182507 | HM190652 | HM191140 | HM190764 | HM190892 | HM190342 | HM182755 | HM182835 | HM182371 |
| A615_35 | HM182377 | HM190499 | HM191190 | HM190696 | HM190886 | HM190326 | HM182719 | HM182784 | HM182367 |
| A615_37 | HM182391 | HM190546 | HM191177 | HM190708 | HM190943 | HM190473 | HM182692 | HM182813 | HM182365 |
| A615_39 | HM182417 | HM190674 | HM191054 | HM190697 | HM190959 | HM190341 | HM182723 | HM182876 | HM182363 |
| A615_62 | HM182440 | HM190662 | HM191127 | HM190772 | HM190944 | HM190335 | HM182724 | HM182873 | HM182361 |
| A615_65 | HM182509 | HM190567 | HM191061 | HM190698 | HM190951 | HM190474 | HM182757 | HM182779 | HM182375 |
| B621_1 | HM182508 | HM190515 | HM191082 | HM190745 | HM190974 | HM190348 | HM182693 | HM182908 | HM182359 |
| B621_10 | HM182434 | HM190601 | HM191173 | HM190686 | HM190977 | HM190397 | HM182717 | HM182899 | HM182358 |
| B621_2 | HM182439 | HM190505 | HM191174 | HM190733 | HM190863 | HM190471 | HM182725 | HM182787 | HM182357 |
| B621_3 | HM182492 | HM190571 | HM191052 | HM190711 | HM190872 | HM190426 | HM182729 | HM182870 | HM182356 |
| B621_4 | HM182418 | HM190530 | HM191222 | HM190688 | HM191016 | HM190384 | HM182758 | HM182910 | HM182355 |
| B621_6 | HM182392 | HM190564 | HM191171 | HM190701 | HM190979 | HM190385 | HM182680 | HM182895 | HM182353 |
| B621_71 | HM182516 | HM190602 | HM191098 | HM190689 | HM190935 | HM190386 | HM182698 | HM182786 | HM182351 |
| B621_72 | HM182528 | HM190557 | HM191201 | HM190792 | HM190912 | HM190398 | HM182645 | HM182920 | HM182350 |
| B621_74 | HM182387 | HM190612 | HM191058 | HM190712 | HM190908 | HM190478 | HM182694 | HM182917 | HM182349 |
| B621_77 | HM182415 | HM190513 | HM191187 | HM190750 | HM191022 | HM190349 | HM182681 | HM182916 | HM182347 |
| B621_78 | HM182419 | HM190572 | HM191172 | HM190687 | HM190978 | HM190409 | HM182759 | HM182948 | HM182346 |
| B621_80 | HM182530 | HM190531 | HM191146 | HM190727 | HM190970 | HM190455 | HM182682 | HM182793 | HM182344 |
| B621_83 | HM182490 | HM190568 | HM191084 | HM190841 | HM190947 | HM190380 | HM182620 | HM182800 | HM182342 |
| B621_87 | HM182519 | HM190605 | HM191212 | HM190754 | HM190988 | HM190410 | HM182655 | HM182822 | HM182339 |
| B621_89_1 | HM182517 | HM190584 | HM191087 | HM190690 | HM190931 | HM190411 | HM182660 | HM182785 | HM182338 |
| B621_90 | HM182518 | HM190594 | HM191097 | HM190695 | HM191007 | HM190412 | HM182716 | HM182782 | HM182335 |
| B621_93 | HM182420 | HM190592 | HM191101 | HM190749 | HM190952 | HM190390 | HM182653 | HM182816 | HM182332 |
| B621_94 | HM182421 | HM190684 | HM191145 | HM190842 | HM190980 | HM190394 | HM182625 | HM182804 | HM182331 |
| H608_1 | HM182422 | HM190563 | HM191167 | HM190759 | HM190992 | HM190429 | HM182753 | HM182819 | HM182330 |
| H608_10 | HM182502 | HM190593 | HM191152 | HM190762 | HM190997 | HM190407 | HM182663 | HM182836 | HM182329 |
| H608_2 | HM182504 | HM190581 | HM191155 | HM190743 | HM190993 | HM190432 | HM182732 | HM182824 | HM182328 |
| H608_3 | HM182423 | HM190640 | HM191194 | HM190739 | HM190933 | HM190363 | HM182751 | HM182931 | HM182327 |
| H608_4 | HM182451 | HM190562 | HM191198 | HM190738 | HM190939 | HM190393 | HM182670 | HM182928 |  |
| H608_41 | HM182424 | HM190558 | HM191162 | HM190756 | HM190991 | HM190447 | HM182675 | HM182826 | HM182325 |
| **Table S2 (continued). GenBank accession numbers for the gene sequences of *T. uzonensis* strains analyzed by MLSA** | | | | | | | | | |
| ***T. uzonensis* strain** | ***gyrB*** | ***rplB*** | ***pyrG*** | ***recG*** | ***recA*** | ***rpoB*** | ***lepA*** | ***leuS*** | **16S rRNA** |
| H608_42 | HM182441 | HM190627 | HM191199 | HM190734 | HM190881 | HM190430 | HM182664 | HM182941 | HM182324 |
| H608_6 | HM182400 | HM190608 | HM191164 | HM190760 | HM191000 | HM190449 | HM182745 | HM182820 | HM182321 |
| H608_71 | HM182467 | HM190630 | HM191165 | HM190761 | HM190996 | HM190362 | HM182731 | HM182827 | HM182320 |
| H608_72 | HM182491 | HM190611 | HM191156 | HM190746 | HM191001 | HM190368 | HM182661 | HM182831 | HM182319 |
| H608_77 | HM182487 | HM190632 | HM191200 | HM190740 | HM190920 | HM190408 | HM182662 | HM182942 | HM182317 |
| H608_78 | HM182466 | HM190542 | HM191053 | HM190723 | HM190940 | HM190450 | HM182602 | HM182943 | HM182316 |
| H608_79 | HM182485 | HM190503 | HM191186 | HM190713 | HM190925 | HM190469 | HM182750 | HM182789 |  |
| H608_81 | HM182472 | HM190574 | HM191123 | HM190718 | HM190879 | HM190472 | HM182730 | HM182838 | HM182314 |
| H608_84 | HM182501 | HM190643 | HM191154 | HM190757 | HM190985 | HM190366 | HM182736 | HM182833 | HM182313 |
| H608_85 | HM182475 | HM190565 | HM191161 | HM190753 | HM190999 | HM190424 | HM182667 | HM182829 | HM182312 |
| H608_88 | HM182412 | HM190540 | HM191166 | HM190747 | HM191002 | HM190459 | HM182668 | HM182828 | HM182310 |
| H608_91 | HM182438 | HM190538 | HM191196 | HM190741 | HM190885 | HM190392 | HM182746 | HM182938 | HM182308 |
| H608_93 | HM182482 | HM190638 | HM191122 | HM190724 | HM190976 | HM190420 | HM182615 | HM182912 | HM182307 |
| I502_40 | HM182471 | HM190670 | HM191128 | HM190771 | HM190883 | HM190314 | HM182747 | HM182799 | HM182305 |
| I502_41 | HM182478 | HM190526 | HM191143 | HM190767 | HM190954 | HM190307 | HM182672 | HM182805 |  |
| I502_42 | HM182500 | HM190655 | HM191131 | HM190773 | HM190914 | HM190317 | HM182709 | HM182855 | HM182304 |
| I502_43 | HM182380 | HM190656 | HM191134 | HM190768 | HM190960 | HM190319 | HM182654 | HM182849 |  |
| I502_45 | HM182474 | HM190654 | HM191137 | HM190777 | HM190950 | HM190320 | HM182699 | HM182843 | HM182302 |
| I502_46 | HM182436 | HM190660 | HM191126 | HM190778 | HM191017 | HM190316 | HM182744 | HM182867 | HM182301 |
| I502_48 | HM182484 | HM190668 | HM191133 | HM190769 | HM190893 | HM190311 | HM182687 | HM182850 |  |
| I502_49 | HM182430 | HM190661 | HM191142 | HM190776 | HM190981 | HM190327 | HM182710 | HM182854 | HM182299 |
| J614_60 | HM182550 | HM190506 | HM191181 | HM190794 | HM190871 | HM190470 | HM182715 | HM182936 | HM182290 |
| J614_61 | HM182554 | HM190517 | HM191178 | HM190795 | HM190921 | HM190493 | HM182714 | HM182944 | HM182289 |
| J614_62_1 | HM182531 | HM190679 | HM191208 | HM190726 | HM190904 | HM190401 | HM182647 | HM182907 |  |
| J614_63 | HM182540 | HM190524 | HM191179 | HM190790 | HM190899 | HM190486 | HM182674 | HM182926 | HM182286 |
| J614_65 | HM182555 | HM190504 | HM191182 | HM190781 | HM190910 | HM190487 | HM182640 | HM182945 | HM182284 |
| J614_7 | HM182524 | HM190588 | HM191204 | HM190692 | HM190963 | HM190400 | HM182648 | HM182903 | HM182283 |
| J614_9 | HM182529 | HM190596 | HM191217 | HM190725 | HM190941 | HM190404 | HM182649 | HM182887 | HM182281 |
| O629_40 | HM182497 | HM190666 | HM191216 | HM190815 | HM191044 | HM190440 | HM182629 | HM182879 | HM182266 |
| O629_42 | HM182401 | HM190539 | HM191151 | HM190720 | HM191023 | HM190494 | HM182638 | HM182871 | HM182264 |
| O629_43 | HM182444 | HM190659 | HM191202 | HM190817 | HM191042 | HM190460 | HM182630 | HM182922 | HM182263 |
| **Table S2 (continued). GenBank accession numbers for the gene sequences of *T. uzonensis* strains analyzed by MLSA** | | | | | | | | | |
| ***T. uzonensis* strain** | ***gyrB*** | ***rplB*** | ***pyrG*** | ***recG*** | ***recA*** | ***rpoB*** | ***lepA*** | ***leuS*** | **16S rRNA** |
| O629_44 | HM182498 | HM190675 | HM191210 | HM190818 | HM190918 | HM190423 | HM182633 | HM182914 | HM182262 |
| O629_45 | HM182511 | HM190676 | HM191209 | HM190810 | HM190958 | HM190370 | HM182623 | HM182900 | HM182261 |
| O629_47 | HM182390 | HM190554 | HM191148 | HM190721 | HM190898 | HM190495 | HM182752 | HM182859 | HM182259 |
| O629_48 | HM182428 | HM190599 | HM191150 | HM190719 | HM191043 | HM190308 | HM182639 | HM182790 | HM182258 |
| O629_49 | HM182496 | HM190677 | HM191207 | HM190811 | HM191030 | HM190418 | HM182631 | HM182924 | HM182257 |
| O629_50 | HM182407 | HM190671 | HM191214 | HM190814 | HM190875 | HM190367 | HM182632 | HM182901 | HM182256 |
| O629_51 | HM182525 | HM190521 | HM191218 | HM190717 | HM190965 | HM190489 | HM182642 | HM182918 | HM182255 |
| O629_6 | HM182470 | HM190658 | HM191203 | HM190829 | HM190969 | HM190360 | HM182624 | HM182915 | HM182254 |
| O629_7 | HM182432 | HM190669 | HM191221 | HM190819 | HM190916 | HM190421 | HM182614 | HM182913 | HM182253 |
| T515_1 | HM182544 | HM190648 | HM191169 | HM190730 | HM190946 | HM190359 | HM182689 | HM182841 | HM182237 |
| T515_10 | HM182376 | HM190629 | HM191193 | HM190736 | HM191015 | HM190433 | HM182691 | HM182934 | HM182236 |
| T515_3 | HM182542 | HM190645 | HM191170 | HM190731 | HM190906 | HM190431 | HM182641 | HM182851 | HM182234 |
| T515_40 | HM182396 | HM190559 | HM191168 | HM190744 | HM190989 | HM190465 | HM182734 | HM182830 | HM182233 |
| T515_41 | HM182493 | HM190585 | HM191078 | HM190702 | HM190949 | HM190436 | HM182700 | HM182886 | HM182232 |
| T515_42 | HM182447 | HM190583 | HM191192 | HM190735 | HM190936 | HM190389 | HM182735 | HM182932 | HM182231 |
| T515_44 | HM182378 | HM190603 | HM191079 | HM190728 | HM190889 | HM190451 | HM182701 | HM182839 | HM182229 |
| V634_1 | HM182477 | HM190606 | HM191094 | HM190704 | HM190876 | HM190427 | HM182741 | HM182905 | HM182226 |
| V634_10_2 | HM182551 | HM190509 | HM191180 | HM190791 | HM190972 | HM190481 | HM182619 | HM182933 | HM182224 |
| V634_2 | HM182473 | HM190604 | HM191089 | HM190714 | HM190903 | HM190466 | HM182671 | HM182888 | HM182223 |
| V634_3 | HM182399 | HM190577 | HM191106 | HM190816 | HM190870 | HM190371 | HM182608 | HM182845 | HM182222 |
| V634_5 | HM182426 | HM190649 | HM191114 | HM190812 | HM190877 | HM190372 | HM182593 | HM182811 | HM182219 |
| V634_6 | HM182386 | HM190607 | HM191092 | HM190715 | HM191034 | HM190373 | HM182737 | HM182906 |  |
| V634_7 | HM182512 | HM190646 | HM191096 | HM190707 | HM190902 | HM190425 | HM182733 | HM182897 | HM182217 |
| V634_71 | HM182406 | HM190636 | HM191107 | HM190827 | HM190890 | HM190415 | HM182590 | HM182862 | HM182215 |
| V634_73 | HM182427 | HM190573 | HM191119 | HM190823 | HM191035 | HM190419 | HM182594 | HM182860 | HM182213 |
| V634_74 | HM182383 | HM190553 | HM191112 | HM190821 | HM191031 | HM190365 | HM182599 | HM182857 | HM182212 |
| V634_77 | HM182398 | HM190548 | HM191120 | HM190830 | HM190955 | HM190374 | HM182600 | HM182797 | HM182211 |
| V634_8 | HM182488 | HM190647 | HM191108 | HM190824 | HM190901 | HM190452 | HM182605 | HM182794 | HM182209 |
| V634_81 | HM182411 | HM190609 | HM191115 | HM190831 | HM190923 | HM190434 | HM182598 | HM182806 | HM182208 |
| V634_82 | HM182409 | HM190587 | HM191113 | HM190813 | HM190874 | HM190422 | HM182592 | HM182856 | HM182207 |
| V634_83 | HM182385 | HM190589 | HM191095 | HM190710 | HM191024 | HM190428 | HM182743 | HM182898 | HM182206 |
| **Table S2 (continued). GenBank accession numbers for the gene sequences of *T. uzonensis* strains analyzed by MLSA** | | | | | | | | | |
| ***T. uzonensis* strain** | ***gyrB*** | ***rplB*** | ***pyrG*** | ***recG*** | ***recA*** | ***rpoB*** | ***lepA*** | ***leuS*** | **16S rRNA** |
| V634_84 | HM182403 | HM190590 | HM191121 | HM190833 | HM190927 | HM190464 | HM182603 | HM182792 | HM182205 |
| V634_85 | HM182429 | HM190631 | HM191093 | HM190705 | HM191046 | HM190369 | HM182763 | HM182889 | HM182204 |
| V634_9 | HM182404 | HM190600 | HM191088 | HM190716 | HM190905 | HM190375 | HM182688 | HM182894 | HM182200 |
| Z606_3 | HM182514 | HM190549 | HM191072 | HM190782 | HM190888 | HM190413 | HM182636 | HM182807 | HM182192 |
| Z606_36 | HM182465 | HM190535 | HM191074 | HM190693 | HM191009 | HM190483 | HM182738 | HM182891 | HM182185 |
| Z606_38 | HM182454 | HM190529 | HM191062 | HM190787 | HM190956 | HM190417 | HM182617 | HM182801 | HM182183 |
| Z606_70 | HM182463 | HM190533 | HM191075 | HM190789 | HM190953 | HM190443 | HM182612 | HM182863 | HM182178 |
| Z606_71 | HM182443 | HM190525 | HM191067 | HM190785 | HM191020 | HM190438 | HM182622 | HM182864 | HM182177 |
| Z606_72 | HM182523 | HM190523 | HM191050 | HM190729 | HM191012 | HM190351 | HM182705 | HM182919 | HM182176 |
| Z606_73 | HM182534 | HM190507 | HM191051 | HM190703 | HM191010 | HM190352 | HM182669 | HM182949 | HM182175 |
| Z606_77 | HM182462 | HM190536 | HM191059 | HM190786 | HM191014 | HM190442 | HM182611 | HM182798 | HM182172 |
| Z606_81 | HM182460 | HM190498 | HM191189 | HM190742 | HM190937 | HM190491 | HM182601 | HM182937 | HM182169 |
| Z606_9 | HM182445 | HM190575 | HM191063 | HM190788 | HM191027 | HM190445 | HM182618 | HM182814 | HM182168 |
